# Examination of sexual health literacy and cyberchondria levels in women with vaginismus

**DOI:** 10.55730/1300-0144.6156

**Published:** 2026-01-10

**Authors:** Gonca TÜRKER ERGÜN, Cemal ÜNLÜ, Ayşe KUYULU AKMAN, Elçin İŞLEK SEÇEN, Raziye TOKSÖZ

**Affiliations:** 1 Department of Obstetrics and Gynecology, Ankara Bilkent City Hospital, Ankara, Turkiye; 2 Department of Obstetrics and Gynecology, Faculty of Medicine, Ankara Yıldırım Beyazıt University, Ankara, Turkiye

**Keywords:** Sexual health literacy, cyberchondria, vaginismus

## Abstract

**Background/aim:**

This study aimed to compare sexual health literacy and cyberchondria levels between women who had been diagnosed with vaginismus and those who had not.

**Materials and Methods:**

The research was conducted as a prospective cohort study including 60 women over the age of 18 who were married and unable to engage in sexual intercourse due to primary vaginismus and 60 sexually active married women without vaginismus. Data collection tools included a patient information form, the Cyberchondria Severity Scale (CSS-12-TR), and the Sexual Health Literacy Scale (SHLS).

**Results:**

The median age of the women diagnosed with vaginismus was 27 (21–54) years, while the median age of the women without a vaginismus diagnosis was 30 (20–57) years. The majority of women diagnosed with vaginismus were not employed and had married for love. No significant difference was found between the groups in terms of the total SHLS score. Women with vaginismus showed lower levels of cyberchondria. Most participants were university graduates and sexual health literacy levels were found to be high.

**Conclusion:**

Cyberchondria levels in women diagnosed with vaginismus may be low because confronting their condition is challenging. However, it can be inferred that women with vaginismus who seek help from healthcare institutions have high levels of problem-solving skills.

## 1. Introduction

Vaginismus is defined as the involuntary contraction of the muscles around the vagina, preventing penetration. In other words, it is persistent and recurrent difficulty experienced by a woman in allowing the entry of a penis or any other object into the vagina, despite wanting to engage in sexual intercourse [1]. Vaginismus is the most common condition among sexual dysfunctions. It is classified as primary when a woman has never had sexual intercourse before and as secondary when a woman has previously engaged in sexual intercourse without symptoms but later experiences difficulties [2]. A study conducted by the Sexual Training, Treatment and Research Association reported that 30%–60% of women in Türkiye experience at least one sexual health issue during their lifetimes, with the most commonly reported condition being the inability to engage in intercourse (i.e., vaginismus) at a rate of 9.2% [3]. Globally, the prevalence of vaginismus is stated to range between 0.4% and 1.7% [4]. Descriptions and evaluations of sexual health problems have recently gained significant importance in the field of women’s health. Individuals need to acquire the necessary knowledge to maintain the quality of their sexual lives, recognize sexual health problems, and facilitate early diagnosis and treatment of these issues [5]. When sexual health issues are examined on a global level, unintentional engagement in risky sexual behaviors during adolescence, unintended pregnancies and abortions, sexually transmitted infections, and incidents of sexual violence and abuse are frequently encountered [6]. In this context, the place, form, and sources through which sexual health information is acquired are important. In societies where sexuality is not discussed due to specific social, cultural, or religious beliefs, women who are about to experience sexual intercourse for the first time are particularly likely to turn to internet sites for information instead of consulting a doctor or healthcare professional [7]. The increasing use of the internet and easy access to technology provide individuals with opportunities to obtain more information about their health and diseases more easily. With the use of the internet as a source for medical information, a new health behavior known as cyberchondria has emerged [8]. Cyberchondria is described as excessive or repeated online searching by individuals to obtain information and alleviate concerns about health problems and diseases [9,10]. It is also defined as the tendency of individuals to self-diagnose or treat themselves by researching health issues they believe they have via the internet. According to a study by the Turkish Statistical Institute, 69.3% of internet users in Türkiye used the internet to search for health-related information [11].

While the internet makes life easier for individuals in many ways, its uncontrolled use can negatively impact individuals both psychologically and socially, leading to excessive health information-seeking behaviors online [12]. When engaging in health information-seeking behaviors, it is crucial for individuals to be able to distinguish whether information is accurate and reliable, highlighting the importance of sexual health literacy levels [13].

This study aims to compare the sexual health literacy and cyberchondria levels of women presenting to our clinic with vaginismus and women without vaginismus.

## 2. Material and methods

This prospective cohort study was conducted in 2024 at Ankara Bilkent City Hospital within the Department of Obstetrics and Gynecology, including the Cosmetic Gynecology and Sexual Dysfunction Clinic and the Gynecology Clinic. Ethical approval was obtained from the Ankara Bilkent City Hospital Clinical Research Ethics Committee (Decision No: TABED 2-24-168; Code No: 168). All participants provided written informed consent prior to enrollment.

The study cohort consisted of women aged 18 years and older who presented to our clinic. The case group included women diagnosed with vaginismus by an experienced researcher (the first author) based on a comprehensive clinical evaluation, including detailed medical and sexual history, and in accordance with the diagnostic criteria of the Diagnostic and Statistical Manual of Mental Disorders, Fifth Edition (DSM-5) [14] and the International Classification of Diseases, Tenth Revision (ICD-10) [15]. The control group comprised married women aged 18 years and older who presented for routine gynecological examination, did not have vaginismus, and agreed to participate in the study.

Women younger than 18 years; those who were unmarried; those with a history of pelvic surgery, neurological disorders, or severe psychiatric illness; those with chronic systemic diseases that may affect sexual function; those who were pregnant or in the postpartum period; and those who were unable to complete the questionnaires or declined to provide informed consent were excluded from the study.

Certain sociodemographic characteristics of the women participating in the study (age, education level, employment status, etc.) were recorded on patient information forms together with information related to their marriages (duration of marriage, spouse’s age, whether the marriage was based on love or courtship, etc.). The Cyberchondria Severity Scale (CSS-12-TR) and the Sexual Health Literacy Scale (SHLS) were used as data collection tools [7,16].

The CSS-12-TR [17] was used to assess the participants’ levels of cyberchondria. The CSS-12-TR is a self-report measurement tool consisting of 12 items. Each item is scored on a scale from 1 to 5 (1: None, 2: Rarely, 3: Sometimes, 4: Often, 5: Always). The scale has four subscales: excessiveness, distress, support-seeking, and compulsion. The total score (minimum of 12, maximum of 60) is calculated by adding the scores for the responses to all items, while subscale scores are obtained by adding the scores for the responses to the items within each subscale. High scores for both the total scale and the subscales provide information about the individual’s level of cyberchondria. In our study, the scale’s Cronbach alpha value was found to be 0.838.

The SHLS, developed by Üstgörül [7], was used to determine the participants’ levels of sexual health literacy. The SHLS consists of 17 questions and comprises two subscales addressing sexual attitude and sexual knowledge. The sexual attitude subscale contains five items, with scores ranging from a minimum of 5 to a maximum of 25. Higher scores for the sexual attitude subscale indicate a negative attitude toward sexual health knowledge. The sexual knowledge subscale includes 12 items, with scores ranging from a minimum of 12 to a maximum of 60. In this study, the Cronbach alpha value for the scale was found to be 0.864.

Researchers administered these questionnaires to both the vaginismus patients and the control group on a face-to-face basis.

### 2.1. Statistical analysis

The mean, standard deviation (SD), maximum, and minimum values of the total and subscale scores of the scales used in the study were determined. Cohort statistics were presented together with mean, SD, minimum, and maximum values. A margin of error of 5% (p < 0.05) was applied in evaluating the hypothesis test findings. All analyses were performed using IBM SPSS Statistics 26 (IBM Corp., Armonk, NY, USA). Questionnaires were administered to participants using the face-to-face technique. The study was conducted with a randomly selected group of patients who applied to the Cosmetic Gynecology and Sexual Dysfunction Clinic and the Gynecology Clinic at Ankara Bilkent City Hospital. The Shapiro–Wilk test was used to examine whether the total scale scores conformed to normal distribution. The independent t-test was used for comparisons of two groups with normal distribution, while the Mann–Whitney U test was used for nonnormally distributed data. A significance level of p < 0.05 was considered in the evaluation of the data. The sample size was calculated at a 95% confidence level and determined to be 120.

## 3. Results

After selection based on the predefined inclusion and exclusion criteria, a total of 120 women were enrolled in the study, including 60 women with vaginismus in the case group and 60 women without vaginismus in the control group. The median age of the case group was found to be 27 (21–54) years, while the median age of the control group was 30 (20–57) years (p = 0.002). Among the women diagnosed with vaginismus, 51.5% were unemployed and 58.0% had love-based marriages. When analyzed by type of marriage, it was observed that 58% of women in love-based marriages had vaginismus. Additionally, a majority of the women in the case group were 35 years old or younger and had university-level education or higher (Table 1).

No significant difference was found between the groups for total mean SHLS score. However, both groups were observed to have high scores. A statistically significant difference was observed between the groups for CSS-12-TR scores (p < 0.05) (Table 2).

The total mean scores and subscale scores of the SHLS for the women participating in the study are shown in Table 2. The mean total SHLS score in the case group was 51.82 ± 11.38, whereas it was 51.37 ± 13.25 in the control group. No statistically significant difference was observed between the groups (p = 0.842).

The mean score for the sexual knowledge subscale of the SHLS was found to be 34.97 ± 10.86 for the case group, while it was 35.95 ± 12.43 for the control group. No statistically significant difference was observed between the groups (p = 0.645). Similarly, no statistically significant difference was observed between the groups for the mean score for the sexual attitude subscale of the SHLS. Women who were employed scored significantly higher on the sexual attitude subscale of the SHLS compared to unemployed women (p = 0.038).

The participants’ mean total scores and subscale scores for the CSS-12-TR are provided in Table 3. The mean total CSS-12-TR score for the case group was 33.73 ± 9.52, while it was 39.52 ± 9.33 for the control group. Thus, the control group had significantly higher total CSS-12-TR scores compared to the case group (p = 0.001).

In the assessment of the excessiveness subscale of the CSS-12-TR, higher total scores were observed in the control group compared to the case group, as well as among women who had love-based marriages. For the support-seeking and compulsion subscales, the mean scores were again higher in the control group than in the case group. In addition, Pearson correlation analysis revealed no significant correlation between the two subscales (p = 0.179).

## 4. Discussion

Accessing services for the healthy development and maintenance of sexual health behaviors is an individual right. Vaginismus, a problem that is frequently encountered in recent years, is viewed as a condition that is hidden by women yet negatively affects their quality of life. Vaginismus is an issue that requires a holistic approach and should be addressed by a multidisciplinary team [18,19].

In treatment and support processes for vaginismus, healthcare professionals should provide services without imposing their own value judgments or sociocultural perspectives on their patients. It is important to conduct an initial evaluation, including the taking of sexual history, that prioritizes privacy and respect and avoids the use of slang or excessive medical information, so that women with vaginismus feel more comfortable. Inadequate knowledge of sexual issues among nurses and other healthcare professionals can lead to difficulties in taking sexual histories or assessing sexuality and may negatively affect patients’ treatment processes [20].

In this study, the median age of the women with vaginismus was 27 (21–54) years, and it was observed that they were typically university graduates. Some studies conducted on this subject have reported similar demographic findings, such as a study in Türkiye conducted with 241 women with vaginismus with a mean age of 30 ± 8.2 years [21], a cross-sectional study from Iran with a mean age of 27.77 ± 5.36 years [22], and another study conducted in Türkiye with the information-motivation-behavior model that included 34 women with vaginismus and a mean age of 27.59 ± 5.32 years [23]. In the present study, the fact that women with vaginismus were generally under the age of 35 could be related to marital age. Additionally, it was found in our study that women with vaginismus over the age of 35 sought hospital care due to concerns about childbearing and disruptions in other areas of life involving factors such as respect, emotional connection, or family communication that could affect the continuation of their marriages.

Regarding the duration of marriage for women diagnosed with vaginismus, an average duration of 20.85 ± 16.21 months was determined in the present study. In Turkish culture, the first year of marriage is considered a period of adjustment to marriage and living together. Couples who were not under social pressure during this time were observed to postpone seeking treatment from healthcare institutions.

This study determined that the majority of women diagnosed with vaginismus had a university-level education or higher and had married out of love. In the study conducted by Doğan and Saraçoğlu, it was observed that women diagnosed with vaginismus were mostly elementary school graduates and housewives, with marriages based on “acquaintance/understanding/flirting” [24]. In another study, 82% of women diagnosed with vaginismus had university-level or higher education, and 90% had married out of love or after dating [25], which is consistent with our study. With the recent expansion and accessibility of digital information, individuals with university-level education or higher may be more likely to make efforts to find solutions to vaginismus, leading to an increase in the diagnosis rates of this sociodemographic group.

In 2023, a study on the behaviors of women experiencing vaginismus using forum sites in Türkiye concluded that women use digital platforms to search for doctors and gather information about treatment methods [26]. In this regard, evaluating vaginismus patients in terms of disease research and sexual health literacy via the internet is important.

A significant difference was observed in the total CSS-12-TR scores of the women with vaginismus (p=0.001). The group without vaginismus had higher cyberchondria scores. No statistically significant difference was found in total CSS-12-TR scores between the groups based on marriage type or employment status (p > 0.05). Women with vaginismus may have less desire to share and seek information about issues related to sexuality due to fear.

In this study, scores for the excessiveness subscale of the CSS-12-TR were found to be higher in the vaginismus group. In this regard, researching an unexperienced behavior or event via the internet may be more common behavior among women diagnosed with vaginismus. It was concluded that women with vaginismus who had high scores for the excessiveness subscale of the CSS-12-TR exhibited rapidly increasing or repetitive behaviors in their internet searches.

Differences in marriage type and employment status were not observed between the groups in this study. This may be attributed to the similarity of social and moral values between the groups. However, a statistically significant difference was observed between the two groups for the support-seeking subscale of the CSS-12-TR (p < 0.05).

A statistically significant difference was found between the scores of women with and without a diagnosis of vaginismus for the compulsion subscale of the CSS-12-TR (p < 0.05). Internet searches related to chronic illness symptoms or perceived changes in the body may negatively affect other social development processes of the individual, such as following the news or sports, engaging in entertainment, or communicating with friends and family. No statistically significant difference was observed between the groups based on marriage type or employment status (p > 0.05).

Sexual health education is important for ensuring the healthy development of an individual’s sexual identity and preventing the occurrence of sexual problems [27]. There is a need for educational programs that are based on reliable, up-to-date, and sufficient information to promote positive behaviors regarding an individual’s sexual health [28]. Scales should be used to identify the groups to be included in these programs and projects and to assess individual levels of knowledge regarding sexual health [20].

In this study, the SHLS was applied to both groups to assess levels of knowledge and attitudes toward sexuality. It was concluded that both groups had good SHLS scores. In an era in which technology has advanced and the educational levels of women are high, it may be assumed that women have knowledge about sexual health topics. However, it seems that there is insufficient motivation and a lack of appropriate conditions to translate that knowledge into behavior.

No statistically significant difference was found between women with and without a diagnosis of vaginismus for the sexual knowledge subscale of the SHLS (p > 0.05). The twenty-first century can be seen as a period in which access to information has become easier but identifying reliable sources has become more challenging. In this context, in a society with increasing levels of education, one’s competence in accessing accurate information and the level of awareness in developing positive behaviors are of crucial importance. However, no statistically significant difference was found based on marital type or employment status (p > 0.05).

For the sexual attitude subscale of the SHLS, no statistically significant difference was found between women with and without vaginismus (p > 0.05). Regarding employment status, employed women had higher sexual attitude scores, and this difference was statistically significant between the groups (p < 0.05). Women with higher scores for the sexual attitude subscale were found to experience more feelings of shame regarding sexual topics, were unable to engage in the reading of materials with sexual content, preferred to avoid sex-centered conversations, and had difficulty sharing openly when faced with any sexual dysfunction, such as vaginismus.

The strengths of this study include its prospective cohort design and the use of validated measurement tools, which enhanced the methodological rigor of the study. Furthermore, the inclusion of a well-defined control group allowed for meaningful comparisons between women with and without vaginismus. The diagnosis of vaginismus was established using standardized DSM-5 and ICD-10 criteria, increasing the diagnostic reliability. Finally, addressing vaginismus within a multidisciplinary and psychosocial framework has provided valuable insights for clinical practice and future intervention programs.

However, this study also has some limitations that should be considered. The cross-sectional nature of the data limits the ability to draw causal inferences between sexual health literacy and cyberchondria. In addition, the study was conducted in a single clinical setting, which may limit the generalizability of the findings. Finally, the use of self-reported questionnaires may have introduced response bias, particularly given the sensitive nature of sexuality-related topics.

In conclusion, women diagnosed with vaginismus require education and support from healthcare professionals to acquire positive sexual behaviors. It is expected that, with appropriate education and counseling provided by healthcare institutions, women’s self-confidence will increase, allowing them to lead healthier lives. Addressing vaginismus as a couple’s problem rather than just a problem for the woman alone is crucial in maintaining respect for women’s identities and eliminating societal pressures directed at women.

## Figures and Tables

**Table 1:** Comparison of sociodemographic characteristics.

	Control n(%)	Vaginismus n(%)	Total n(%)	p
**Age**				
35 and below	39 (43.3)	51 (56.7)	90 (75.0)	**0.011**
Above 35	21 (70.0)	9 (30.0)	30 (25.0)	
**Marriage type**				
Love	34 (42.0)	47 (58.0)	81 (67.5)	**0.011**
Arranged	26 (66.7)	13 (33.3)	39 (32.5)	
**Education level**				
High School and below	37 (66.1)	19 (33.9)	56 (46.7)	**0.001**
University and above	23 (35.9)	41 (64.1)	64 (53.3)	
**Employment status in an income-generating job**				
Employed	28 (51.9)	26 (48.1)	54 (45.0)	0.714
Unemployed	32 (48.5)	34 (51.5)	66 (55.0)	
**Total (%)**	60 (50.0)	60 (50.0)	120 (100.0)	

Characteristics	Vaginismus	Control
**Average age**	27 (21–54)	30 (20–57)
**Average age of spouse**	31.28 ± 5.95	32.76 ± 11.3
**Average length of marriage (months)**	20.85 ± 16.21	23.87 ± 8.56

* Categorical variables are presented as number (%) and continuous variables as median (minimum–maximum) or mean ± standard deviation; group comparisons were performed using the chi-square (χ^2^) test and p < 0.05 was considered statistically significant.

**Table 2:** Mean total and subscale scores for the Sexual Health Literacy Scale.

Variables	SHL total score (Mean± SD)	p	SHL Sexual knowledge (Mean± SD)	p	SHL Sexual attitude (Mean± SD)	p
**Vaginismus**						
**Exists**	51.82 **±** 11.38	0.842	34.97 **±** 10.86	0.645	16.85 **±** 6.15	0.200
**None**	51.37 **±** 13.25		35.95 **±** 12.43		15.42 **±** 6.03	
**Marriage type**						
**Love**	52.44 **±** 11.83	0.276	35.65 **±** 11.79	0.792	16.79 **±** 6.00	0.090
**Arranged**	49.82 **±** 13.20		35.05 **±** 11.45		14.77 **±** 6.17	
**Employment status**						
**Employed**	52.44 **±** 12.42	0.494	35.04 **±** 11.67	0.721	17.41 **±** 5.72	**0.038**
**Unemployed**	50.89 **±** 12.25		35.80 **±** 11.68		15.09 **±** 6.25	

* Values are expressed as mean ± standard deviation; comparisons between groups were performed using the independent-samples t-test and p < 0.05 was considered statistically significant.

**Table 3:** Mean total and subscale scores for the Cyberchondria Severity Scale.

Variables	n	Total score (mean ± SD)	p	Extremity subcale (mean ± SD)	P	Distress subscale (mean ± SD)	p	Support-seeking subscale (mean ± SD)	p	Compulsion subscale (mean ± SD)	p
**Vaginismus**											
**Exists**	60	33.73 **±** 9.52	**0.001**	10.27 **±** 3.22	**0.004**	8.60 **±** 3.03	0.072	8.77 **±** 3.27	**0.026**	6.10 **±** 3.24	**<0.001**
**None**	60	39.52 **±** 9.33		8.58 **±** 3.12		9.60 **±** 3.00		10.03 **±** 2.90		11.30 **±** 3.23	
**Marriage type**											
**Love**	81	37.19 **±** 9.83	0.371	9.85 **±** 3.43	**0.039**	9.31 **±** 3.06	0.371	9.56 **±** 3.30	0.436	8.47 **±** 4.31	0.382
**Arranged**	39	35.46 **±** 9.85		8.54 **±** 2.95		8.67 **±** 3.00		9.08 **±** 2.77		9.18 **±** 3.80	
**Employment status**											
**Employed**	54	35.81 **±** 10.84	0.426	9.06 **±** 3.18	0.264	8.78 **±** 3.48	0.310	9.20 **±** 3.32	0.538	8.78 **±** 4.10	0.854
**Unemployed**	66	37.29 **±** 8.94		9.73 **±** 3.32		9.36 **±** 2.63		9.56 **±** 3.00		8.64 **±** 4.22	

* Values are expressed as mean ± standard deviation; p-values were obtained using the independent-samples t-test and p < 0.05 was considered statistically significant.

## Data Availability

Data are available upon request from the corresponding author.
